# Supplementary material for: Anion-exchange-mediated internal electric field for boosting photogenerated carrier separation and utilization
Source: Nat Commun. 2021 Aug 16;12:4952. doi: 10.1038/s41467-021-25261-8 (PMC8368037; doi:10.1038/s41467-021-25261-8)
Supplement: Supplementary file 1 — Supplementary Information [file 41467_2021_25261_MOESM1_ESM.pdf]

Supporting Information

Anion-Exchange-Mediated Internal Electric Field for Boosting Photogenerated Carrier Separation and Utilization

Tong Han[1#], Xing Cao[1#], Kaian Sun[1#], Qing Peng[1*], Chenliang Ye[1], Aijian Huang[1], Weng-Chon Cheong[2], Zheng Chen[3], Rui Lin[4], Di Zhao[5], Xin Tan[1], Zewen Zhuang[1], Chen Chen[1*], Dingsheng Wang[1], and Yadong Li[1*]

[1]Department of Chemistry, Tsinghua University, Beijing 100084, China.

[2]Department of Physics and Chemistry, Faculty of Science and Technology, University of Macau, Macao SAR, China.

[3]College of Chemistry and Materials Science, Anhui Normal University, Wuhu 241000, China.

[4]Nanoinstitute Munich, Ludwig-Maximilians-Universität München, 80539, Munich, Germany.

[5]Key Laboratory of Cluster Science, Ministry of Education of China, Beijing Key Laboratory of Photoelectronic/Electrophotonic Conversion Materials, School of Chemistry and Chemical Engineering Beijing Institute of Technology Beijing 100081, China.

[#]These authors contributed equally to this work: Tong Han, Xing Cao, Kaian Sun.

[*]Email: pengqing@mail.tsinghua.edu.cn; cchen@mail.tsinghua.edu.cn; ydli@mail.tsinghua.edu.cn

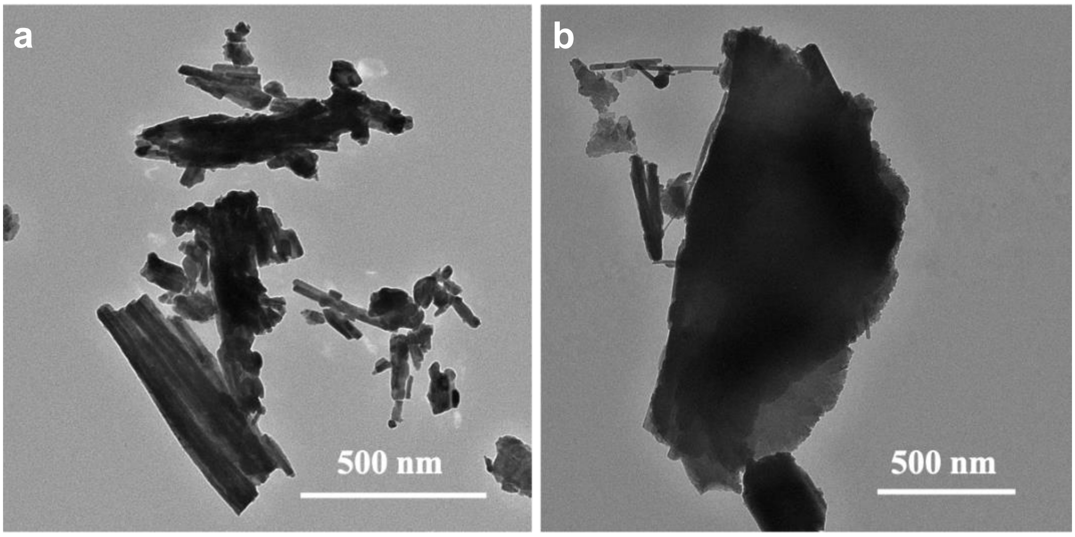

Supplementary Figure 1. Solvothermal reaction products without mannitol or PVP. a TEM image of the hydrothermal product without mannitol. **b** TEM image of the hydrothermal product without PVP.

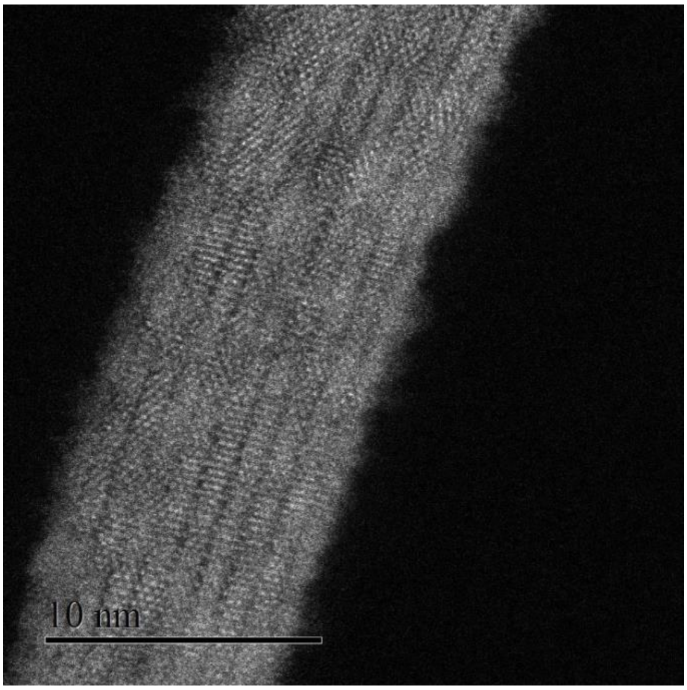

Supplementary Figure 2. Atomic-resolution HAADF-STEM image of BOH.

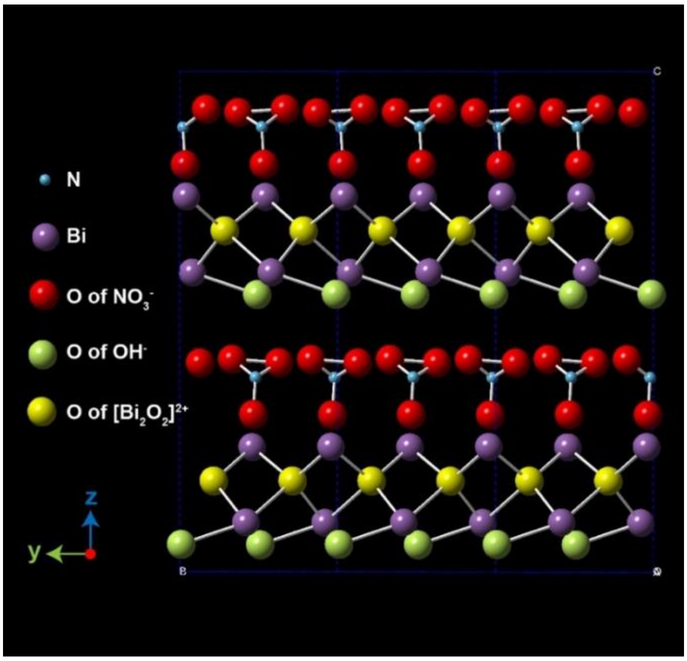

Supplementary Figure 3. Two-dimensional model structure of $Bi_2O_2(OH)(NO_3)$, viewed down the x-axis.

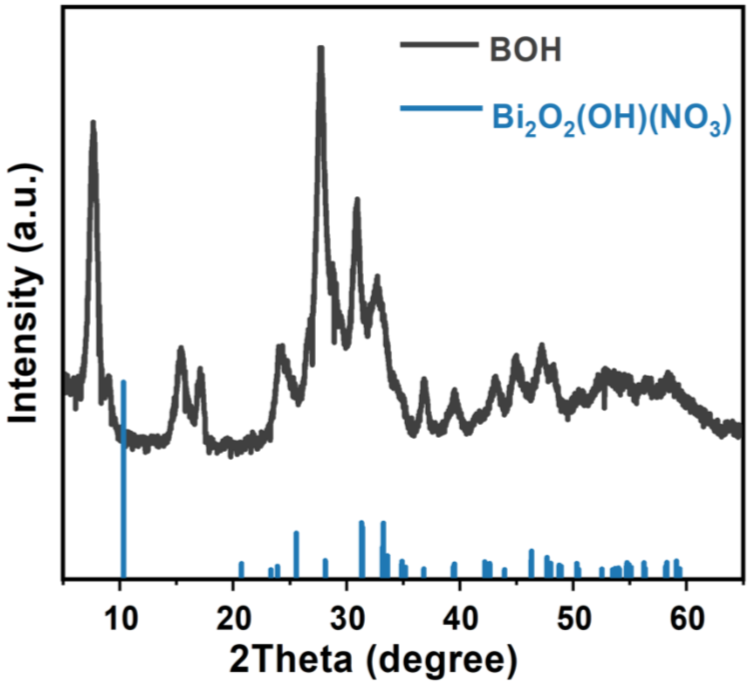

Supplementary Figure 4. The XRD patterns of the BOH and $Bi_2O_2(OH)(NO_3)$.

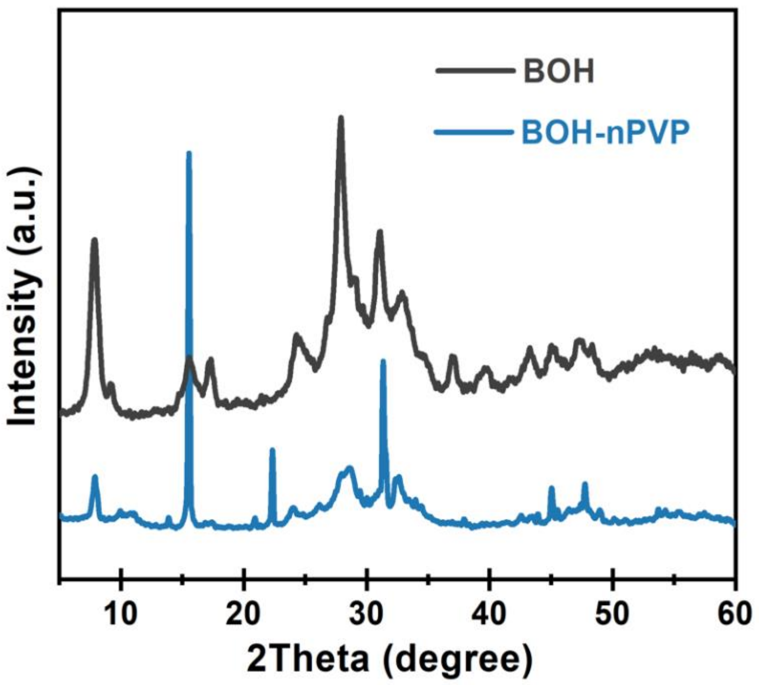

Supplementary Figure 5. The XRD patterns of the BOH and BOH-nPVP.

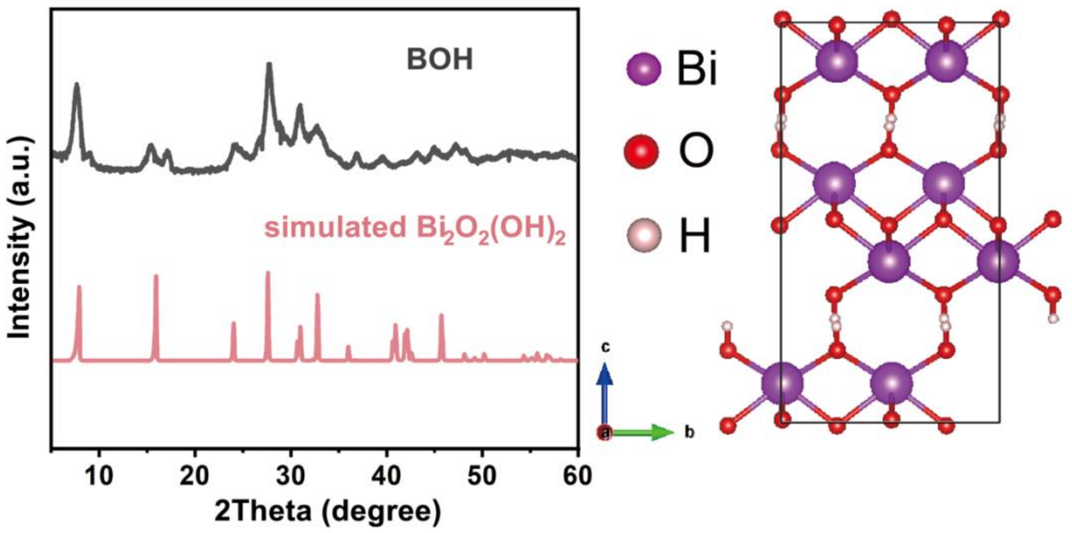

Supplementary Figure 6. The XRD patterns of the BOH and simulated $Bi_2O_2(OH)_2$. For the assumed crystal cell, a=6.74 Å; b=5.80 Å; c=22.20 Å; α=90˚;β=90˚;γ=90˚.

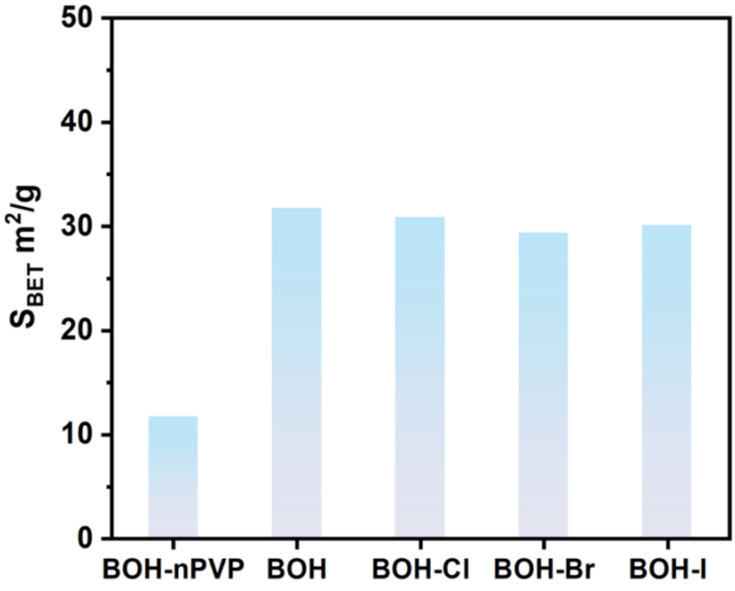

Supplementary Figure 7. The specific surface areas of BOH-nPVP, BOH and BOH-X.

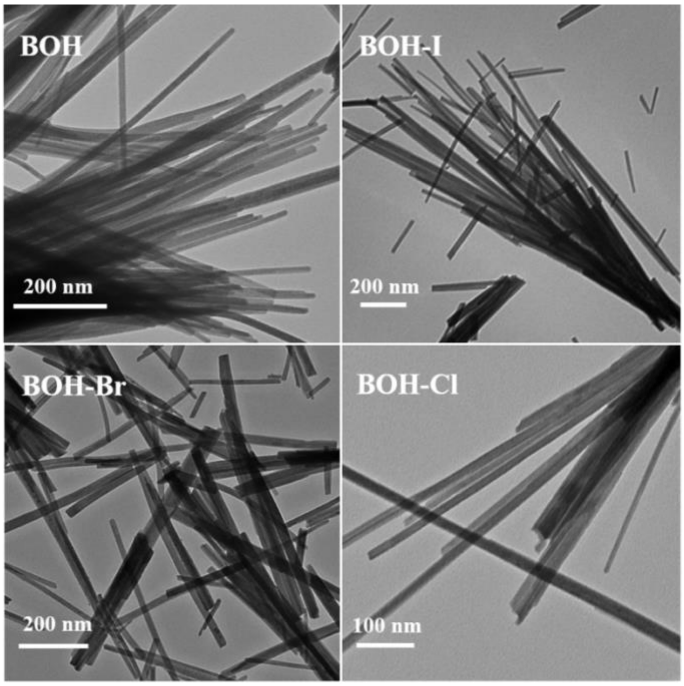

Supplementary Figure 8. TEM images of BOH and BOH-X samples.

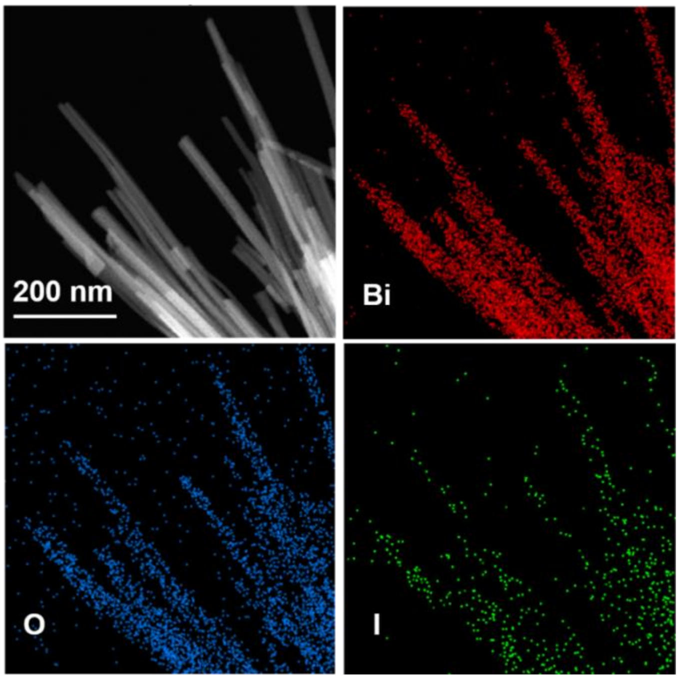

Supplementary Figure 9. The EDS mapping of BOH-I.

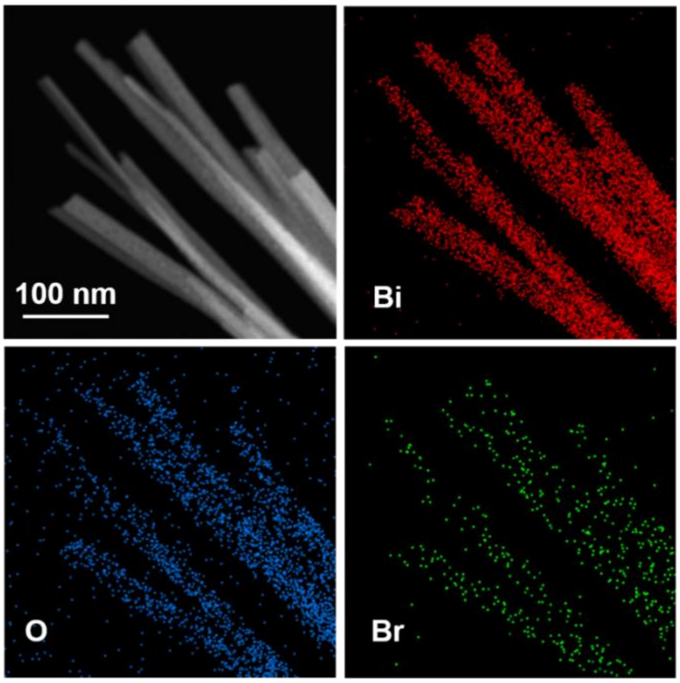

Supplementary Figure 10. The EDS mapping of BOH-Br.

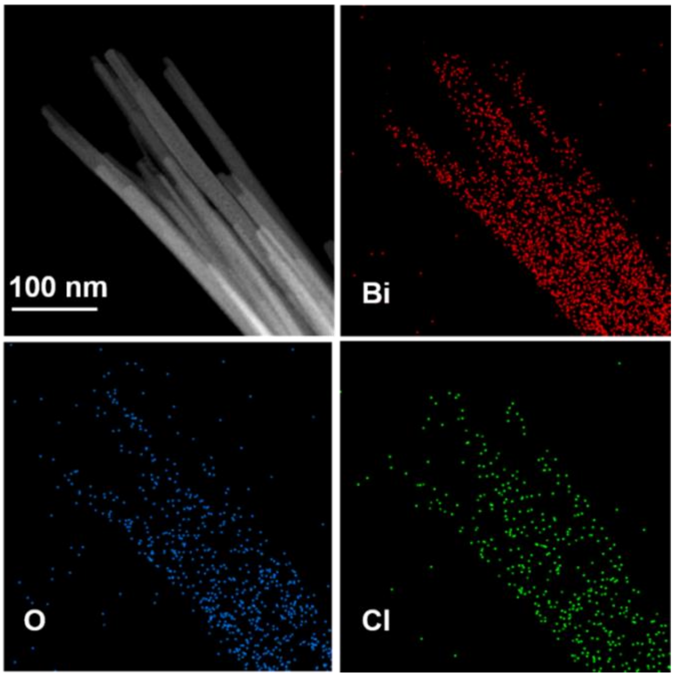

Supplementary Figure 11. The EDS mapping of BOH-Cl.

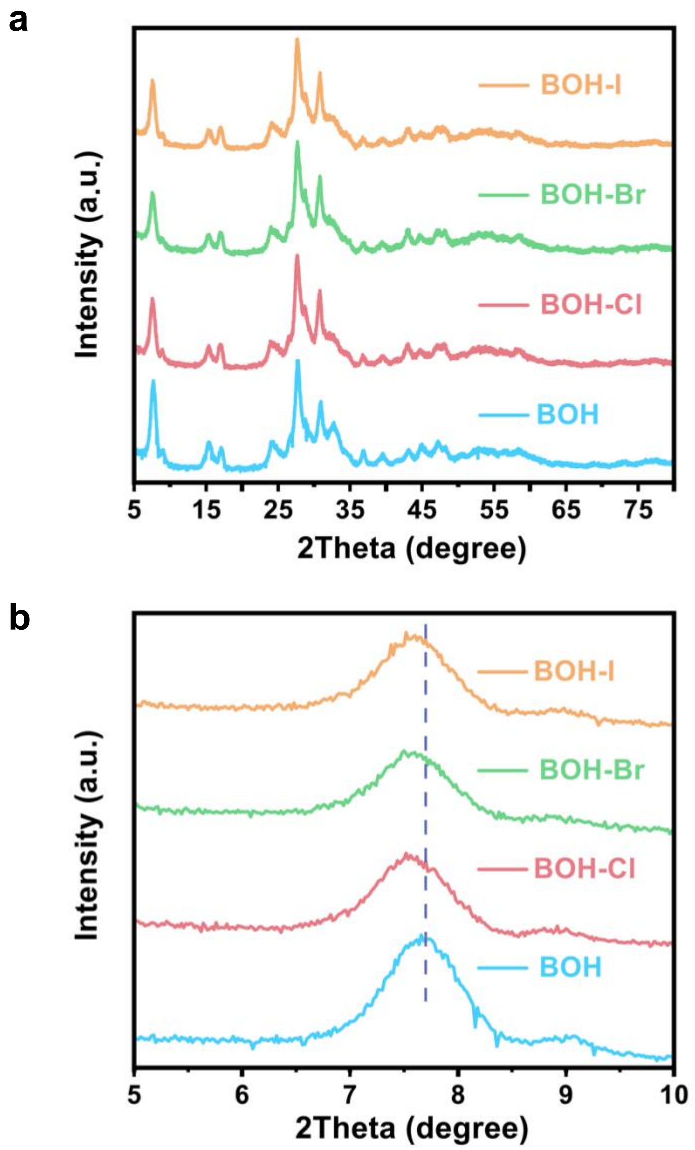

Supplementary Figure 12. The XRD patterns of the BOH and BOH-X. a The normal XRD pattern. **b** The local XRD pattern.

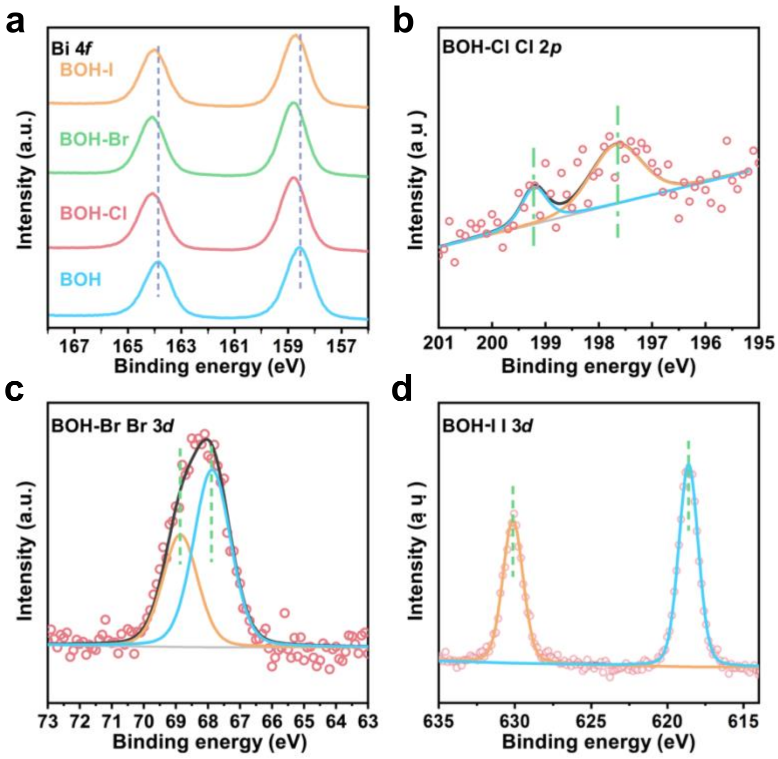

Supplementary Figure 13. XPS of samples for Bi and halogens. a XPS spectra of Bi in the samples before and after ion exchange. **b–d** XPS spectra of halogens in the BOH-Cl, BOH-Br, and BOH-I, respectively.

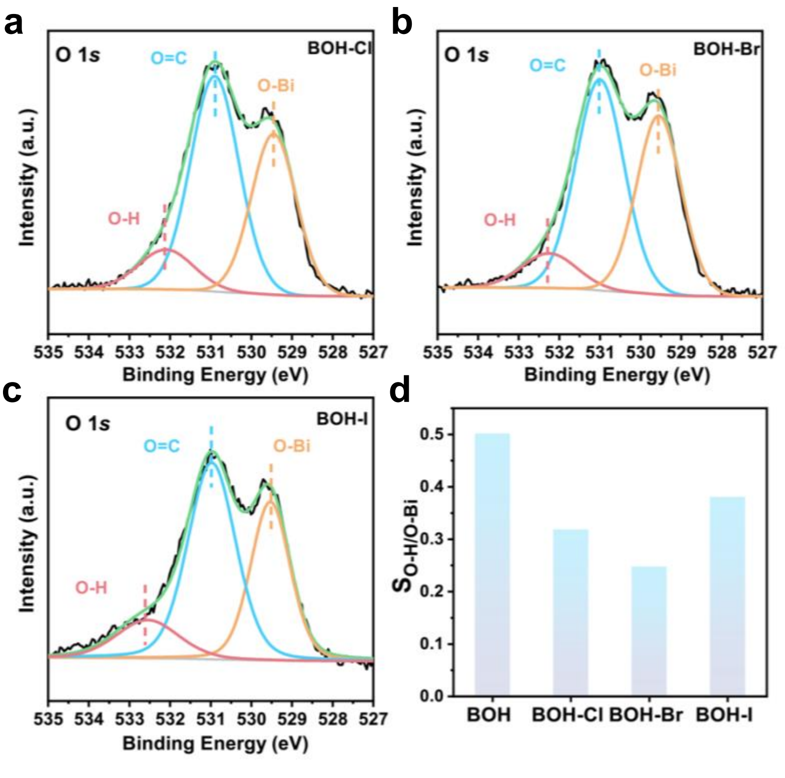

Supplementary Figure 14. Characterization of samples. a–c XPS spectra of O in the ion-exchanged samples. **d** the ratios of O–H bond before and after ion exchange.

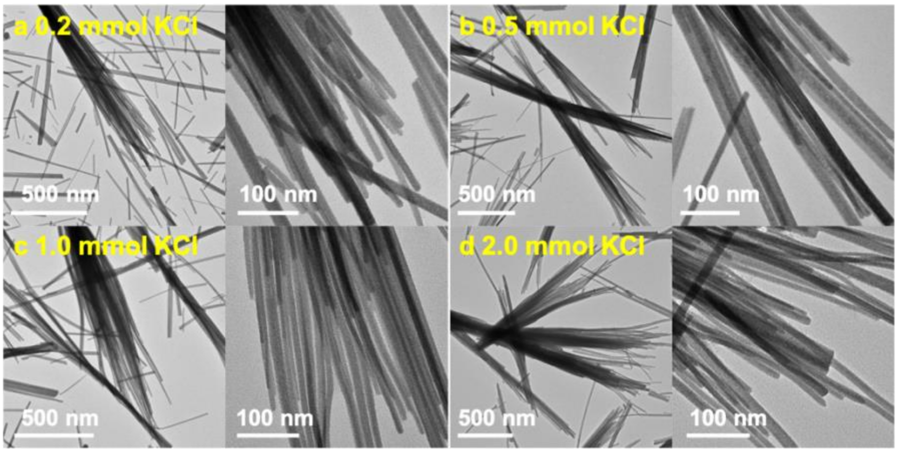

Supplementary Figure 15. TEM characterization of ion-exchange products with different amounts of Cl ions.

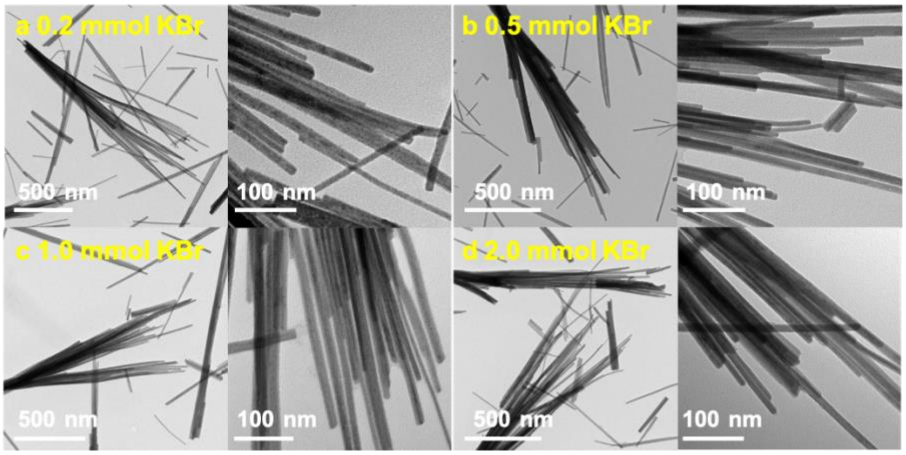

Supplementary Figure 16. TEM characterization of ion-exchange products with different amounts of Br ions.

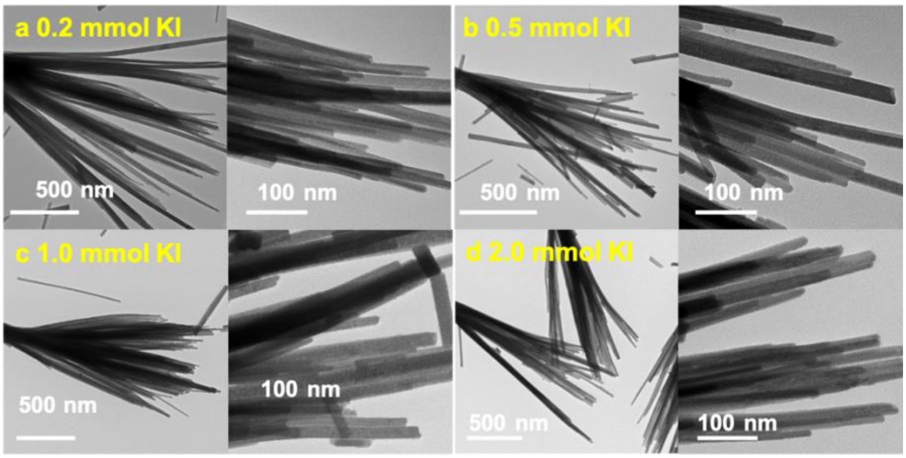

Supplementary Figure 17. TEM characterization of ion-exchange products with different amounts of I ions.

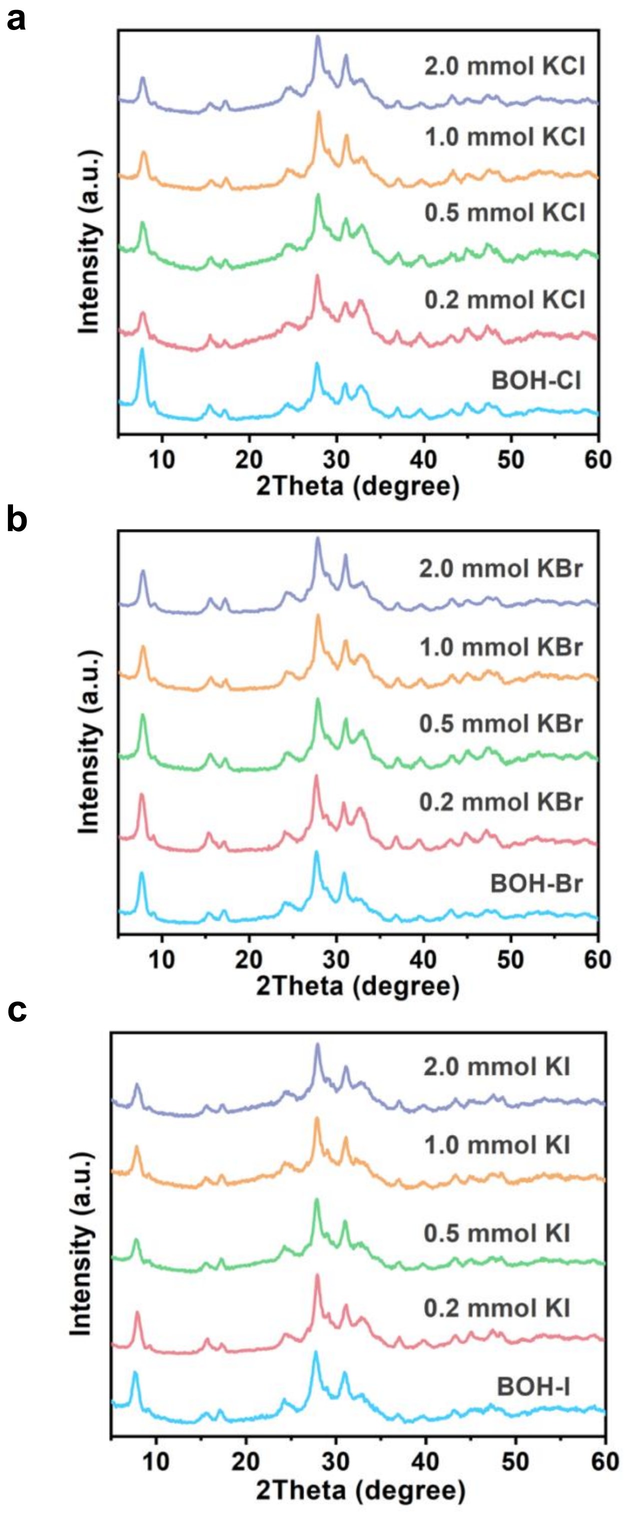

Supplementary Figure 18. Characterization of samples. The XRD characterization of ion-exchange products with increased amounts of Cl^- (**a**), Br^- (**b**), I^- (**c**).

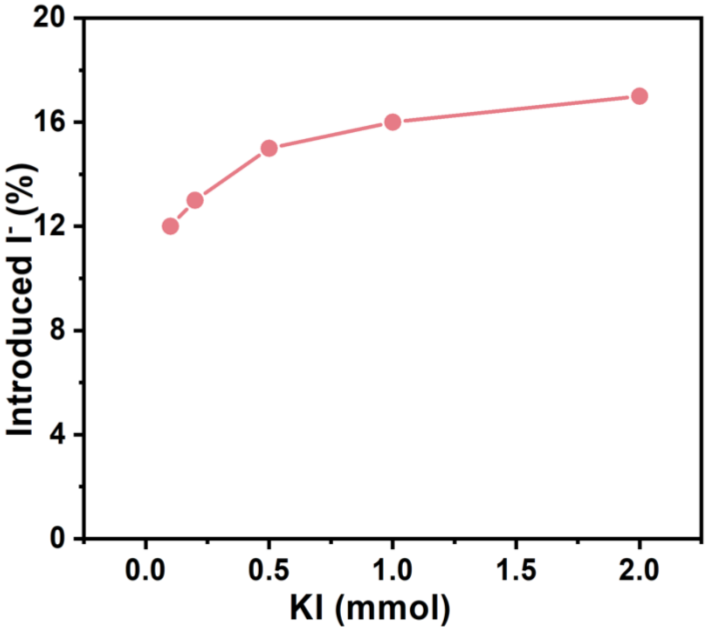

Supplementary Figure 19. The relationship between the amount of KI added in the synthesis and the actual introduction of I^- (from the XPS semi-quantitative experiments).

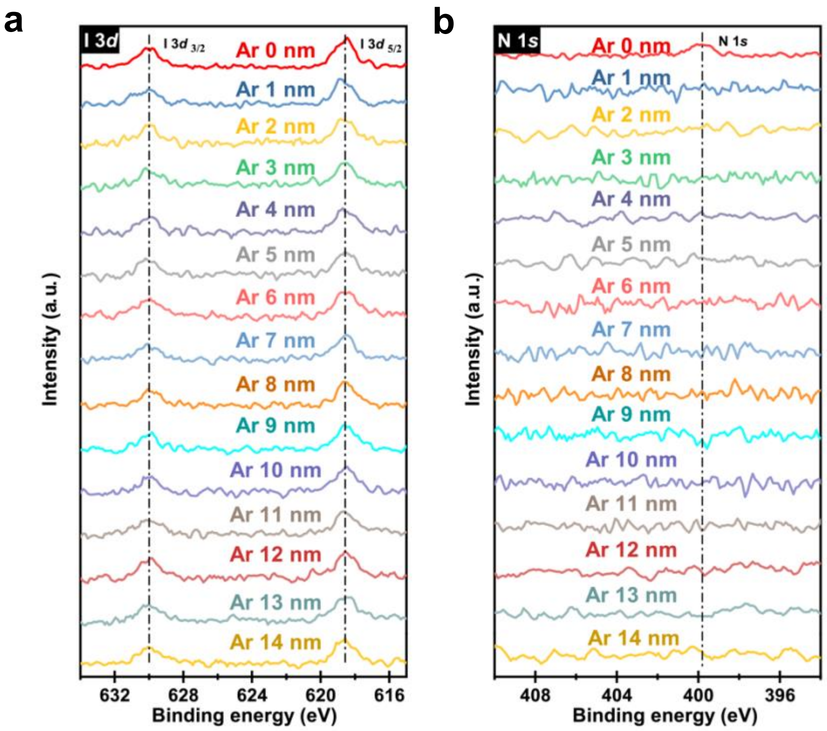

Supplementary Figure 20. Characterization of BOH-I. High-resolution XPS spectra of I 3*d* (**a**) and N 1*s* (**b**) for BOH-I with Ar^+ sputtering at different depths.

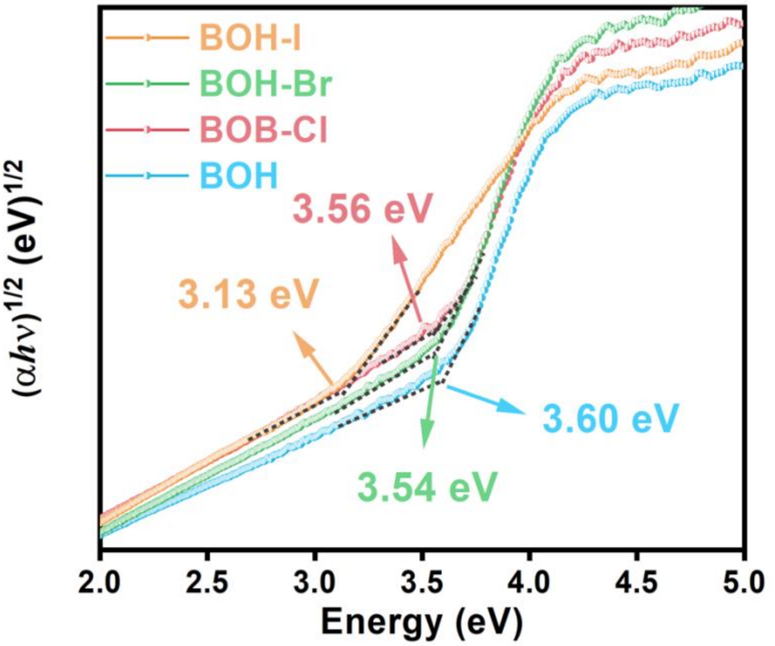

Supplementary Figure 21. Tauc plots of BOH and BOH-X samples.

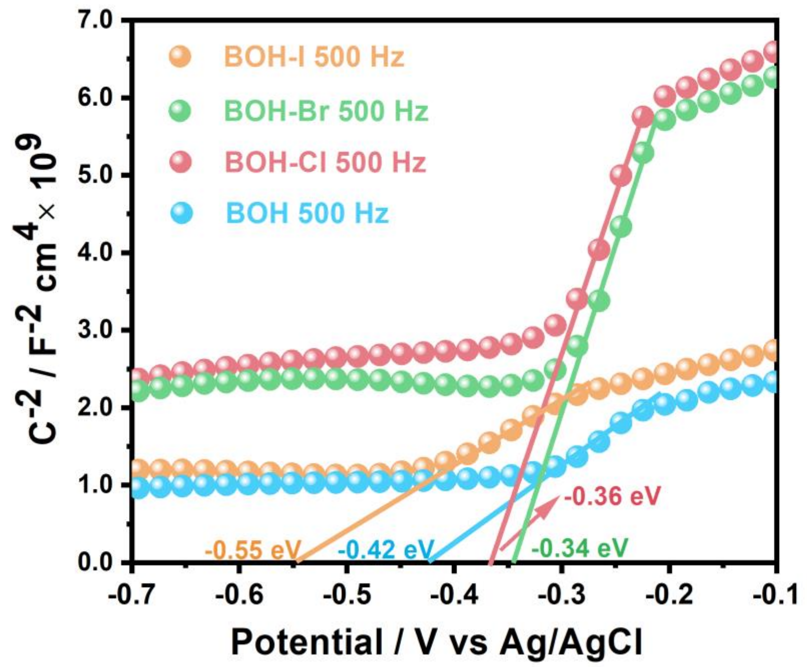

Supplementary Figure 22. Mott–Schottky plots of BOH and BOH-X samples.

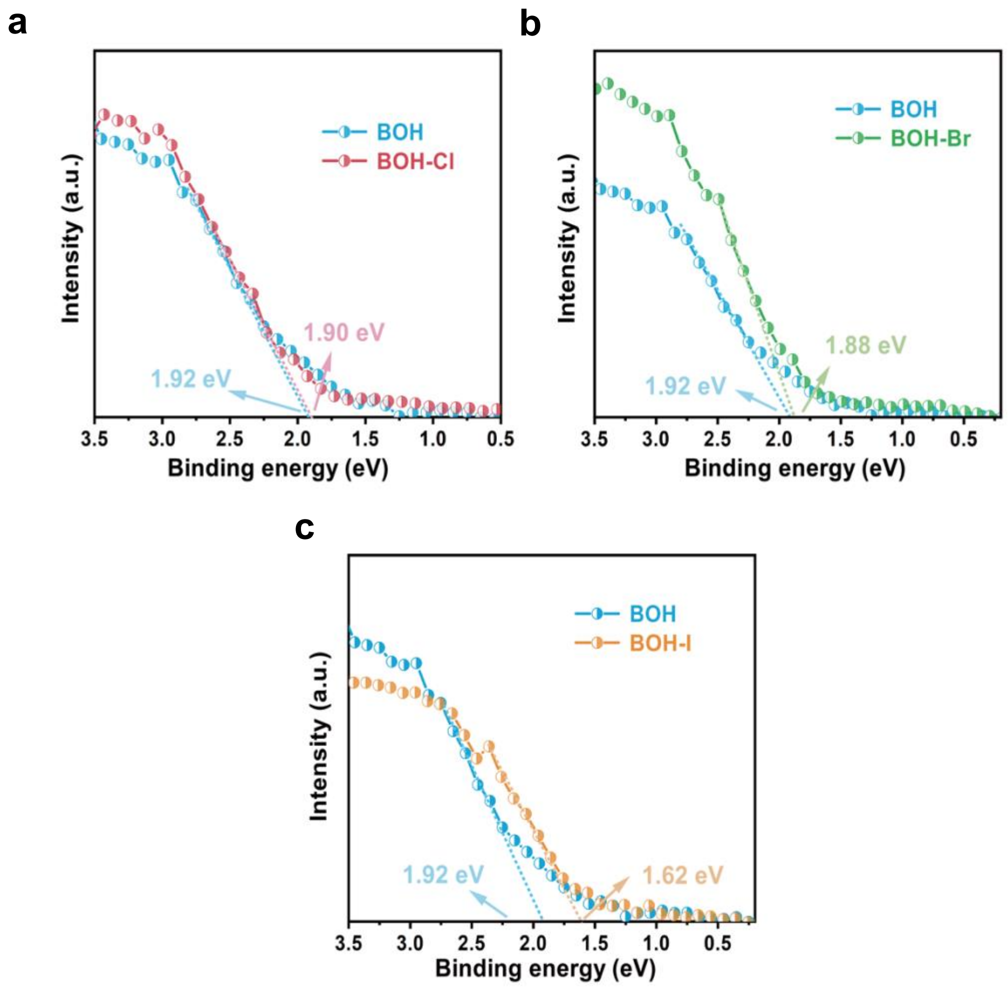

Supplementary Figure 23. The characterization of valence band spectra for (a) BOH, BOH-Cl, (b) BOH-Br and (c) BOH-I.

Correspondingly, the distances from the CBM to the Fermi level are calculated to be 1.68 eV, 1.66 eV, 1.66 eV, and 1.51 eV for BOH, BOH-Cl, BOH-Br, and BOH-I, respectively (using the equation $E_{CB}= E_{g}–E_{VB}$ (1)). To assess the Fermi levels for BOH and BOH-X, we conducted the Mott-Schottky measurements at the potential of -0.1 V to -0.7 V (at a fixed frequency of 500 Hz). Based on the Mott–Schottky formula:

$$C_{sc}^{-1}=2(\Delta\Phi_{sc}–RT/F)\,(q\varepsilon\varepsilon_0N)^{-1} \quad (2)$$

(Where $\Delta\Phi_{sc}=V–V_{fb}$ (3), V_{fb} is the flat band potential, T is the Kelvin temperature, F is the Faraday constant, R is the gas constant, ε and ε_0 are the semiconductor dielectric constant and vacuum dielectric constant, respectively, q is the charge quantity, and N is the doping concentration), we plotted C_{sc}^{-1} versus V (Supplementary Figure 22), and

then obtained the flat bands of BOH, BOH-Cl, BOH-Br and BOH-I through the intercept of the abscissa $V_0=V_{fb}+RT/F$. Therefore, the potentials of BOH, BOH-Cl, BOH-Br and BOH-I are 0.15 eV, 0.21 eV, 0.23 eV and 0.02 eV, (versus Ag/AgCl at pH 6.80) respectively, which are -0.25 eV, -0.19 eV, -0.17 eV and -0.38 eV separately, relative to the normal hydrogen electrode at pH=0 (NHE). Since the Fermi level E_f and V_{fb} have the same value, combined with the valence band spectra and UV-vis DRS results, the energy band diagrams of BOH and BOH-X relative to the normal hydrogen electrode are finally obtained as Supplementary Figure 24).

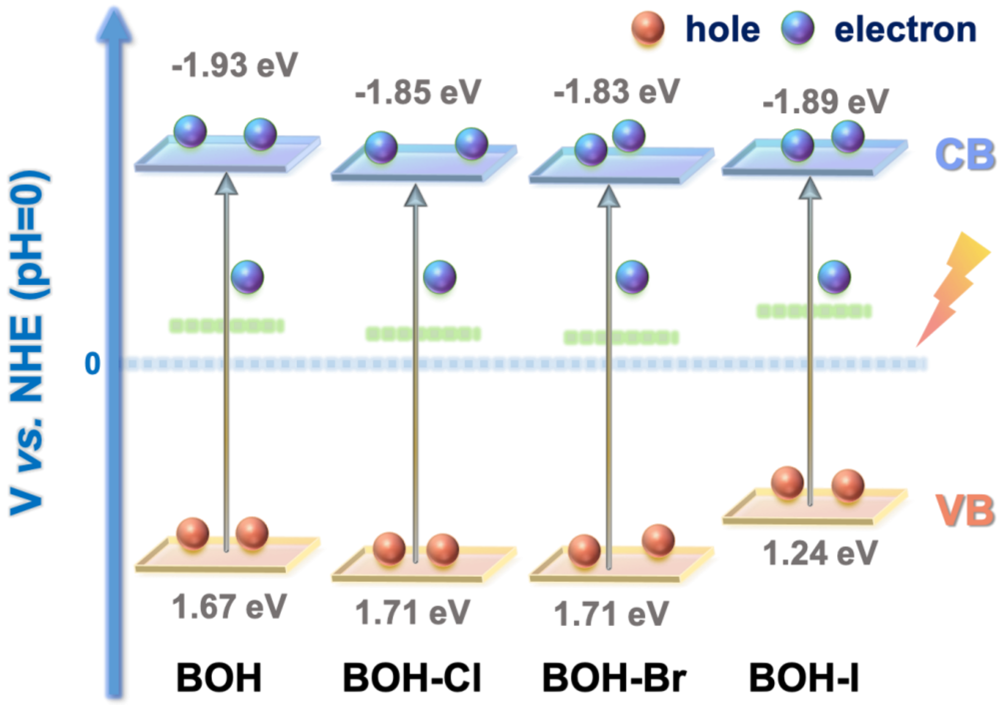

Supplementary Figure 24. The schematic diagram of the band structure of BOH and BOH-X.

To investigate the existence of IEF, we qualitatively measured the internal electric field intensity of BOH and BOH-X samples according to the following equation developed by Kanata et al.

$$F_s=(-2V_s\rho/\varepsilon\varepsilon_0)^{1/2} \quad (1)$$

Where, F_s is the internal electric field magnitude, V_s is the surface voltage, ρ is the surface charge density, Ɛ is the low-frequency dielectric constant, and $Ɛ_0$ is the permittivity of free space. As Ɛ and $Ɛ_0$ are constants, the IEF intensity is determined by the surface voltage (V_s) and the surface charge density (ρ). Therefore, we could qualitatively compare the internal electric field intensity of BOH and BOH-X according to the $(V_s\rho)^{1/2}$ values. Based on that, we first applied the open-circuit potentials measurements to evaluate the surface voltages of BOH and BOH-X. As shown in Supplementary Figure 25, the surface voltage of BOH-I is 0.268 V, which is greater than that of BOH-Br (0.198 V), BOH-Cl (0.175 V), and BOH (0.142 V).

As Le Formal and Gratzel et al. reported, the accumulated positive charge on the surface is proportional to the integral value, which is calculated from the transient photocurrent density minus the steady-state photocurrent density in the same time. Therefore, the transient photocurrent density measurements were conducted. The surface charge densities of BOH and BOH-X were then obtained by the integral of the transient anodic photocurrent peaks (Supplementary Figure 26).) As expected, the integral value of the photocurrent response of BOH-I (148.1μc·cm^{-2}) is the maximum among all specimens, which is even twice as magnitude as that of BOH (70.4 μc ·cm^{-2}), and also higher than that of BOH-Br (123.8 μc·cm^{-2}) and BOH-Cl (105.4 μc·cm^{-2}).

It can be found that the internal electric field intensity of BOH, BOH-Cl, BOH-Br, and BOH-I gradually increased (Figure 4d). And the internal electric field intensity of BOH-I is double BOH's, while BOH-Br and BOH-Cl are 1.6 times and 1.4 times as high as that of BOH, respectively.

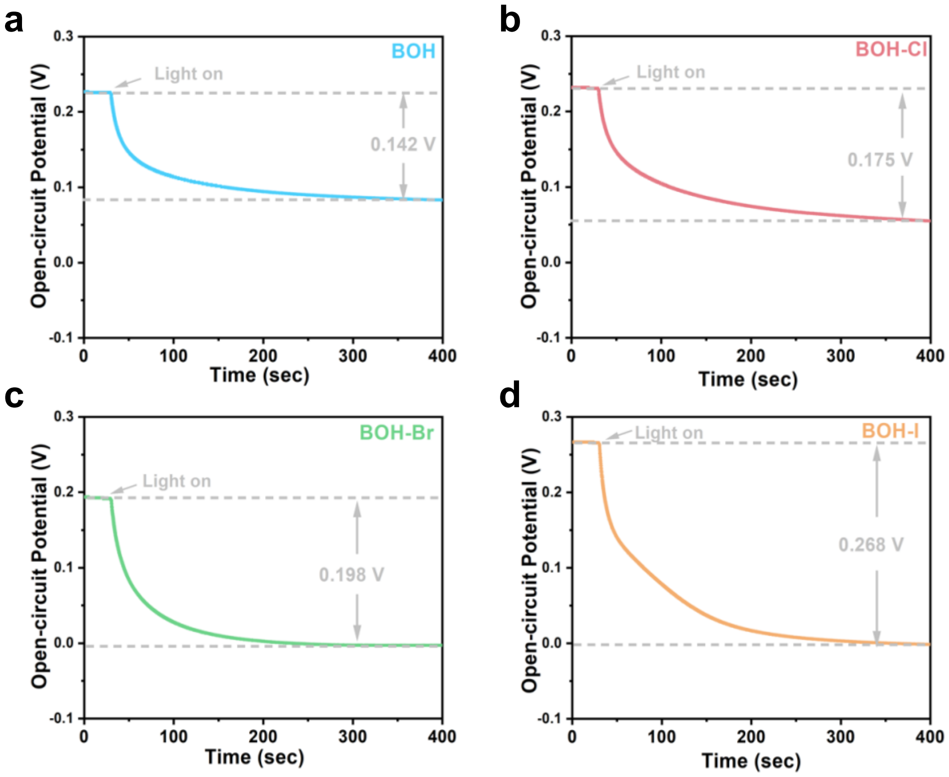

Supplementary Figure 25. The open-circuit potentials of BOH (a) and BOH-X (b–d).

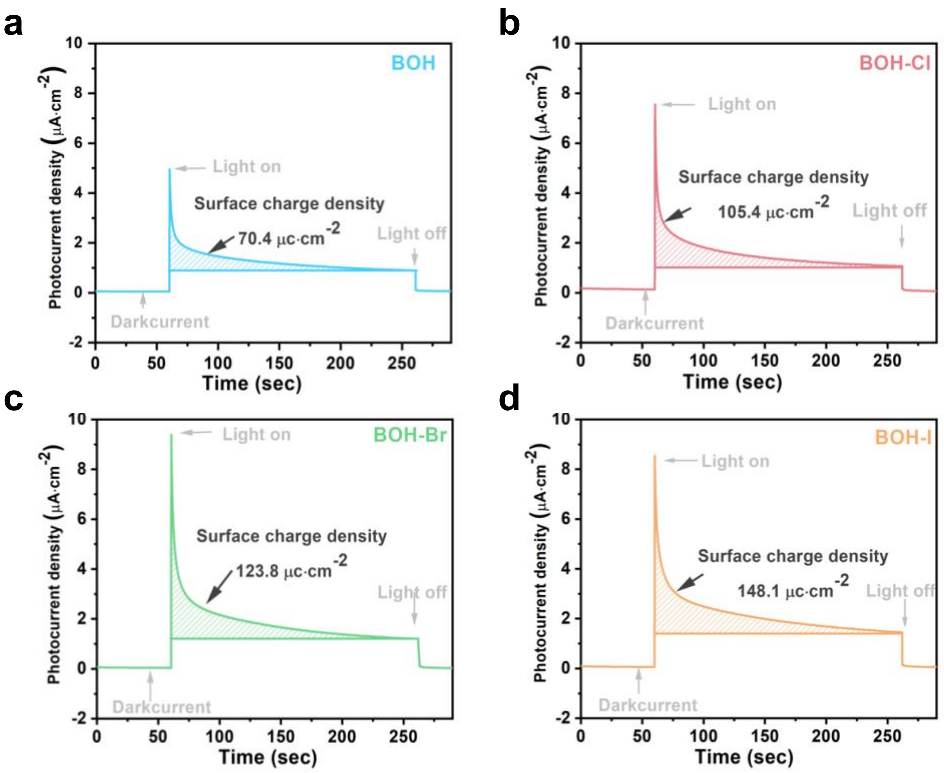

Supplementary Figure 26. The transient photocurrent density of BOH (a) and BOH-X (b–d).

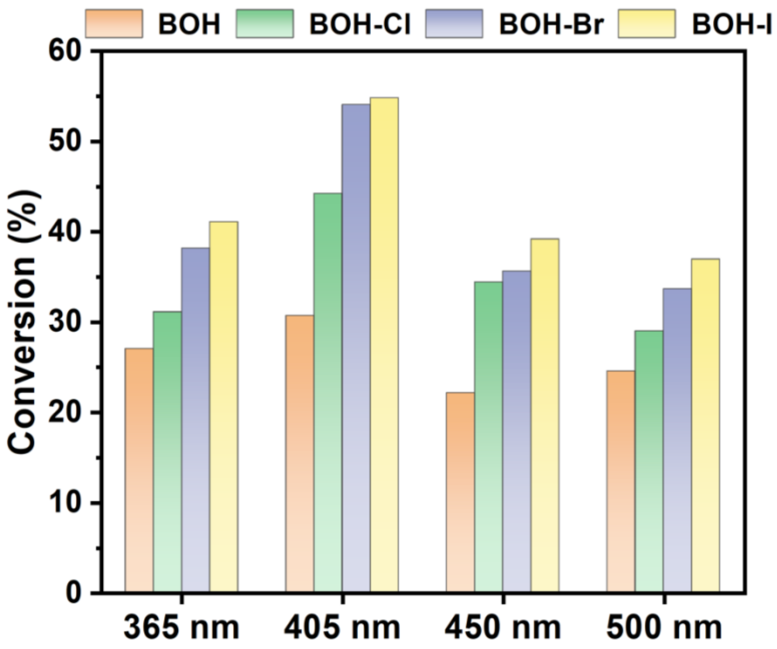

Supplementary Figure 27. Photocatalytic activity over BOH and BOH-X by introducing different single wavelength light.

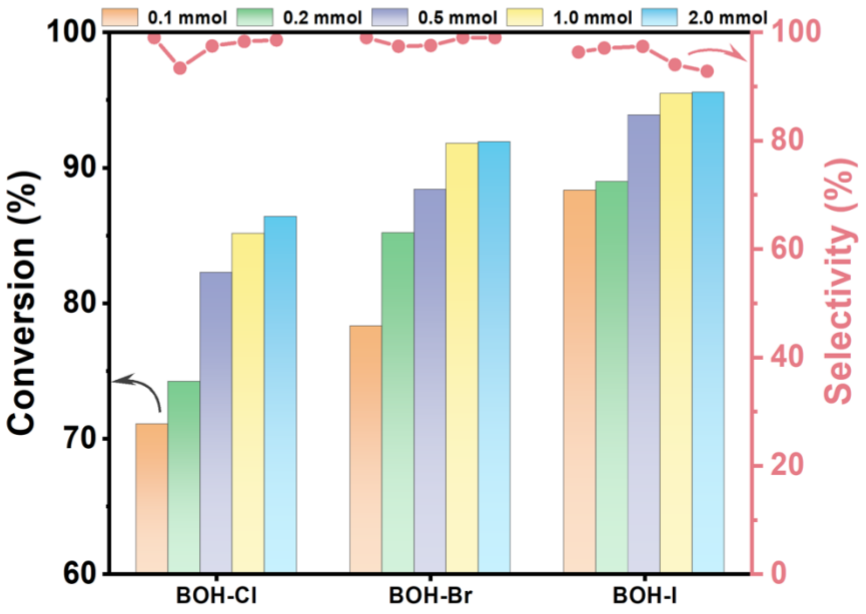

Supplementary Figure 28. The photocatalytic performances of catalysts with more halogen ions.

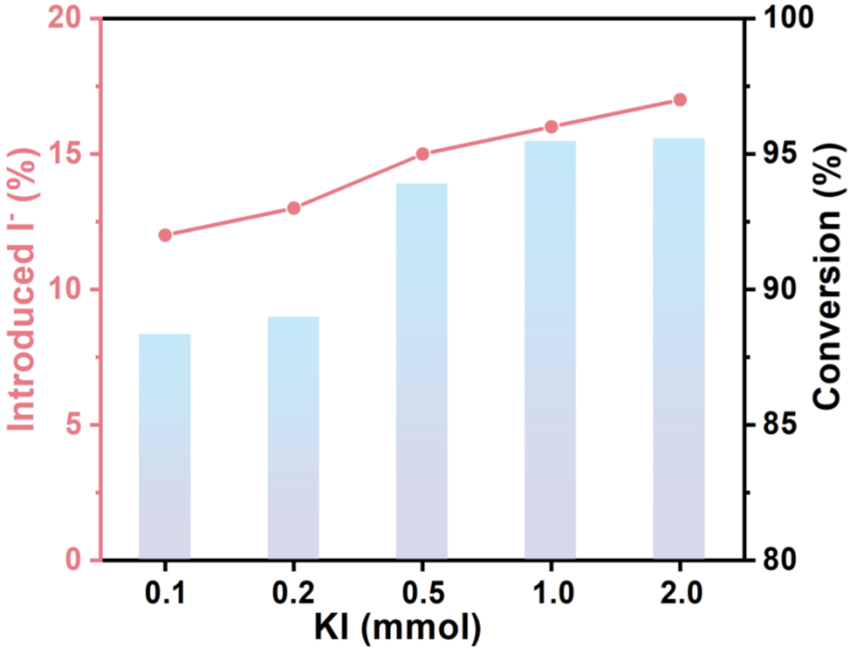

Supplementary Figure 29. The introduction of I^- (the red dots) and the photocatalytic performance (the histogram) plotted versus the amount of KI added in the synthesis.

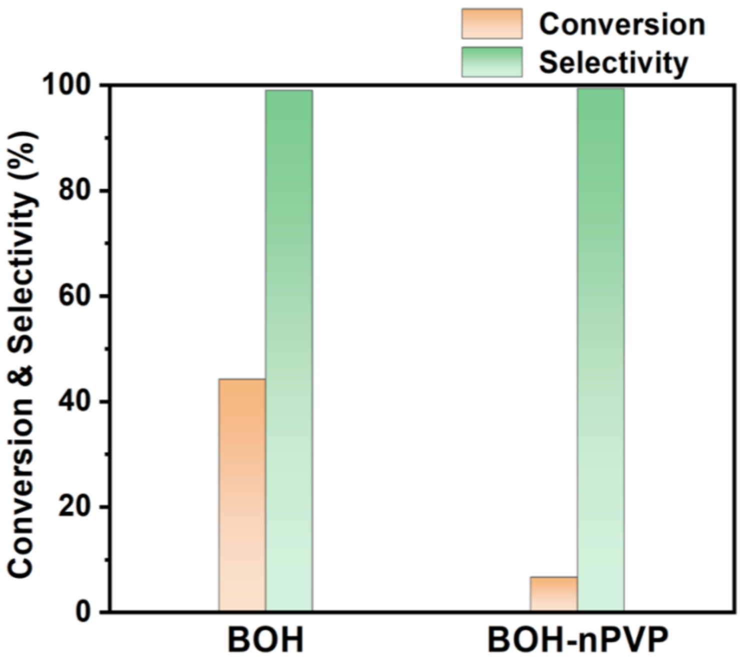

Supplementary Figure 30. Catalytic performances of the normal BOH and BOH-nPVP.

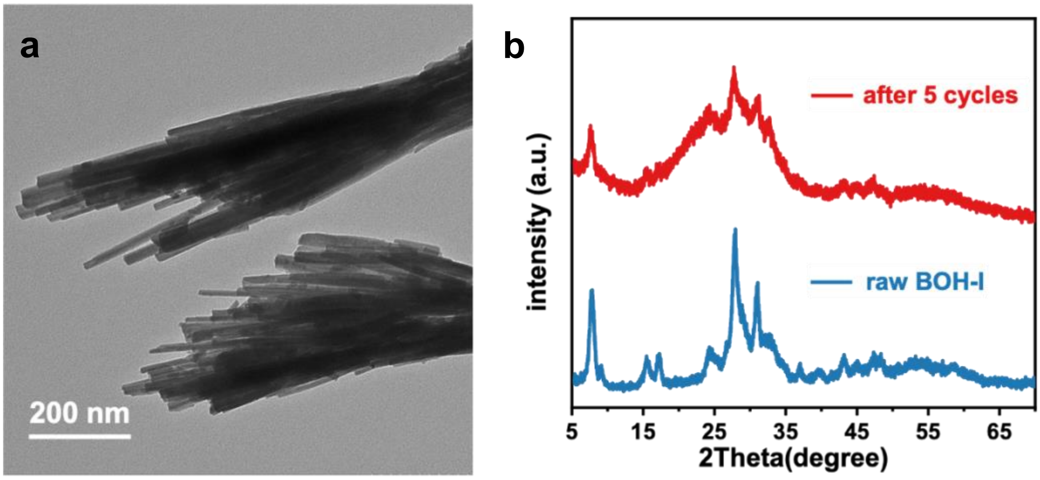

Supplementary Figure 31. Characterization of recycled BOH-I. TEM image (**a**) and XRD patterns (**b**) of BOH-I after five catalytic cycles.

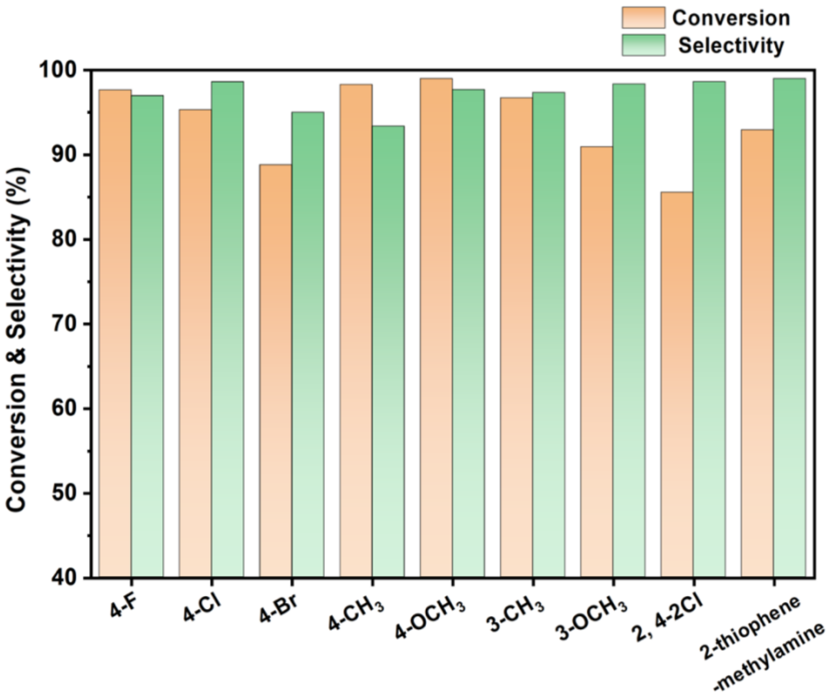

Supplementary Figure 32. Catalytic performance of BOH-I for series of benzylamine derivatives.

Some typical electron sacrificial agents themselves can oxidize benzylamine (Supplementary Figure 33), and different electron scavengers have different quenching effects on benzylamine oxidation (Supplementary Figure 34). Specifically, unlike $K_2S_2O_8$, the addition of CCl_4 promotes the oxidation of benzylamine with BOH, while for BOH-X, the catalytic reactions are inhibited to varying degrees (but less than that of $K_2S_2O_8$). Considering the catalytic performance of CCl_4 and $K_2S_2O_8$, it is believed that electrons also participate in benzylamine oxidation for BOH-X. However, it is difficult to determine whether electrons or holes play a dominant role based on the hindrance of electron and hole sacrificial agents to the catalytic reaction. However, in the photo-oxidation reaction of organic system with oxygen participated, electrons usually participate in the reaction by reducing oxygen to $\cdot O_2^-$. The EPR experiments on superoxide radicals (Supplementary Figure 35) show that all BOH-X could generate more superoxide radicals than BOH. According to the experiment with superoxide radical capturer added (Supplementary Figure 36), the benzylamine conversion was

74.1% using superoxide dismutase (SOD), however, the benzylamine conversion didn't decline significantly. These data support that $\cdot O_2^-$ is less involved in the reaction, hence, for benzylamine photocatalytic oxidation, direct hole oxidation can be considered to play a dominant role to some extent.

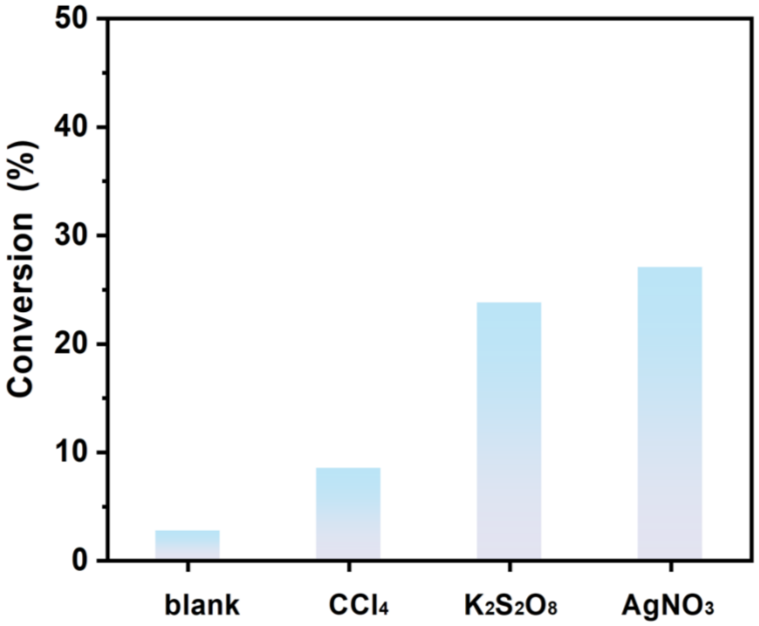

Supplementary Figure 33. The effect of different electronic sacrificial agents on the oxidation reaction of benzylamine (without additional catalyst).

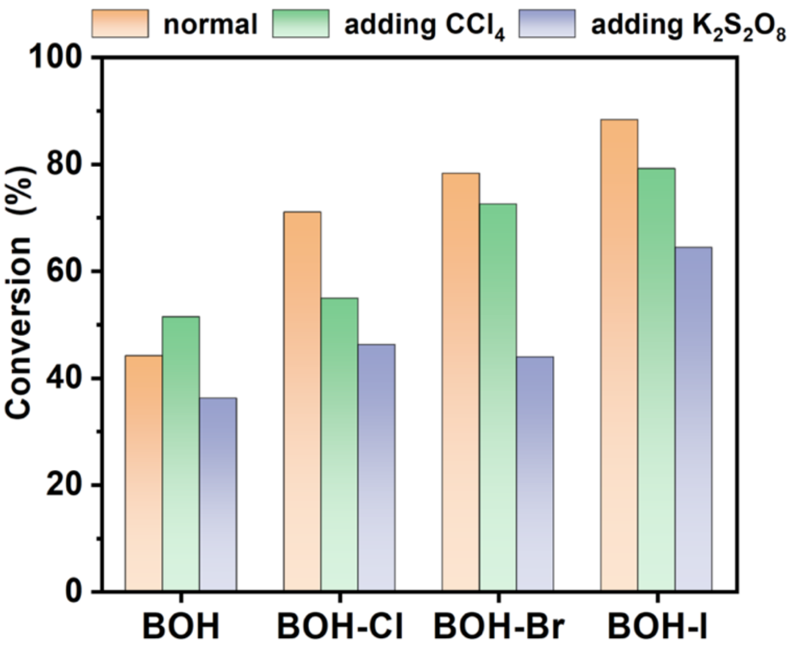

Supplementary Figure 34. The photocatalytic performance of CCl_4 and $K_2S_2O_8$ as electronic sacrificial agents.

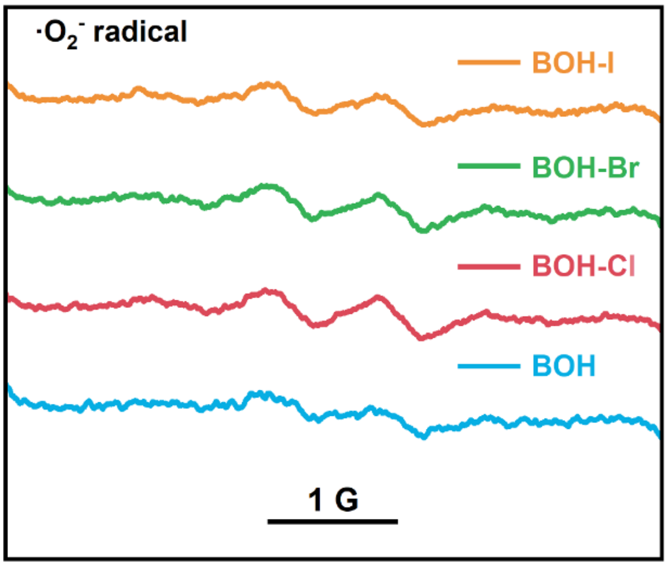

Supplementary Figure 35. EPR spectra for BOH and BOH-X samples.

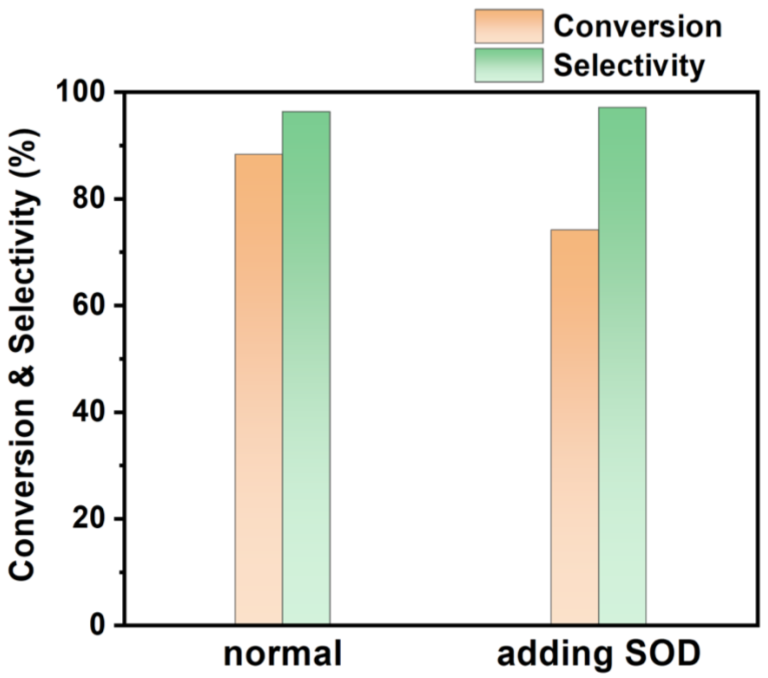

Supplementary Figure 36. Catalytic performances of the normal BOH-I and after addition of superoxide dismutase (SOD).

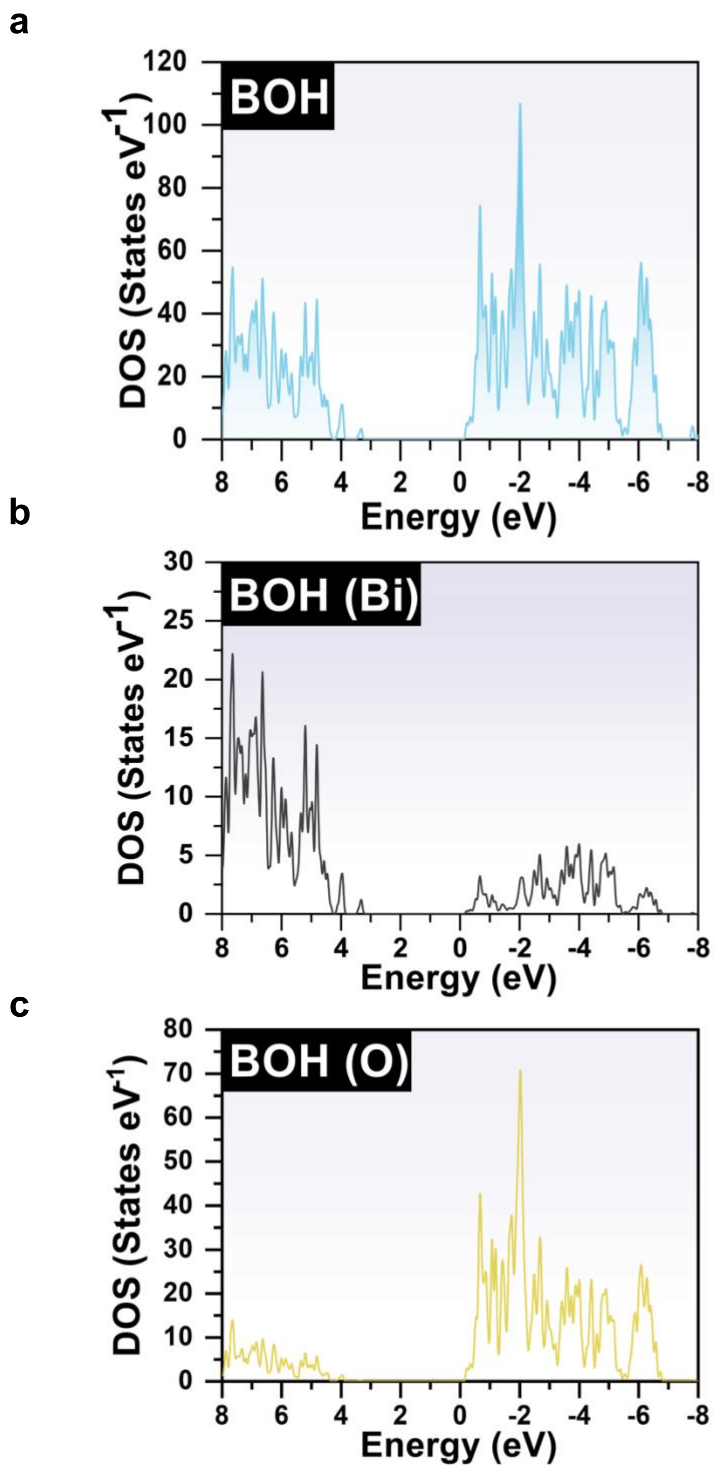

Supplementary Figure 37. The DOS calculation for BOH. a The calculated density of state of BOH. **b** and **c** The calculated partial density of state of bismuth and oxygen element for BOH, respectively.

a

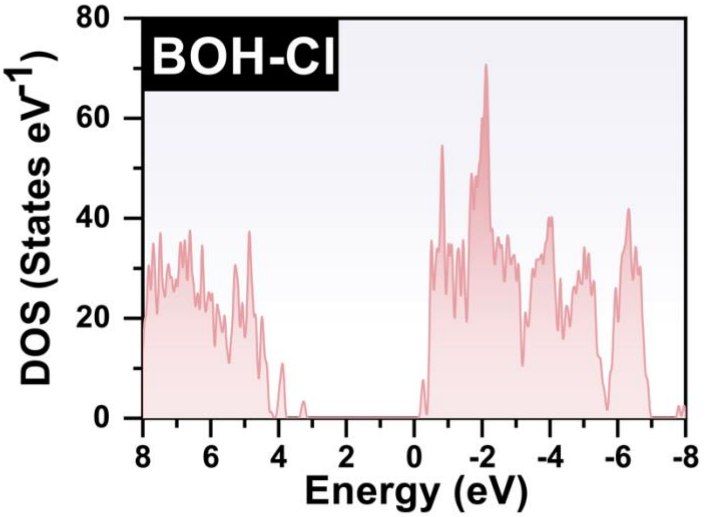

b

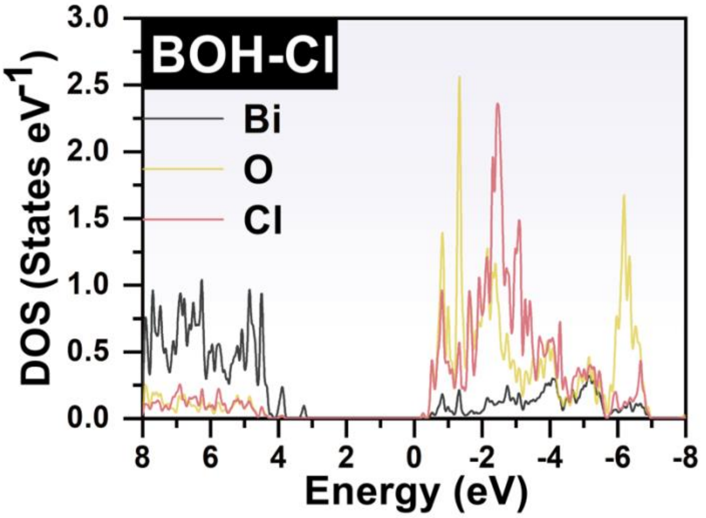

Supplementary Figure 38. The DOS calculation for BOH-Cl. a The calculated density of state of BOH-Cl. **b** The calculated partial density of state of bismuth, oxygen and chlorine element for BOH-Cl, respectively.

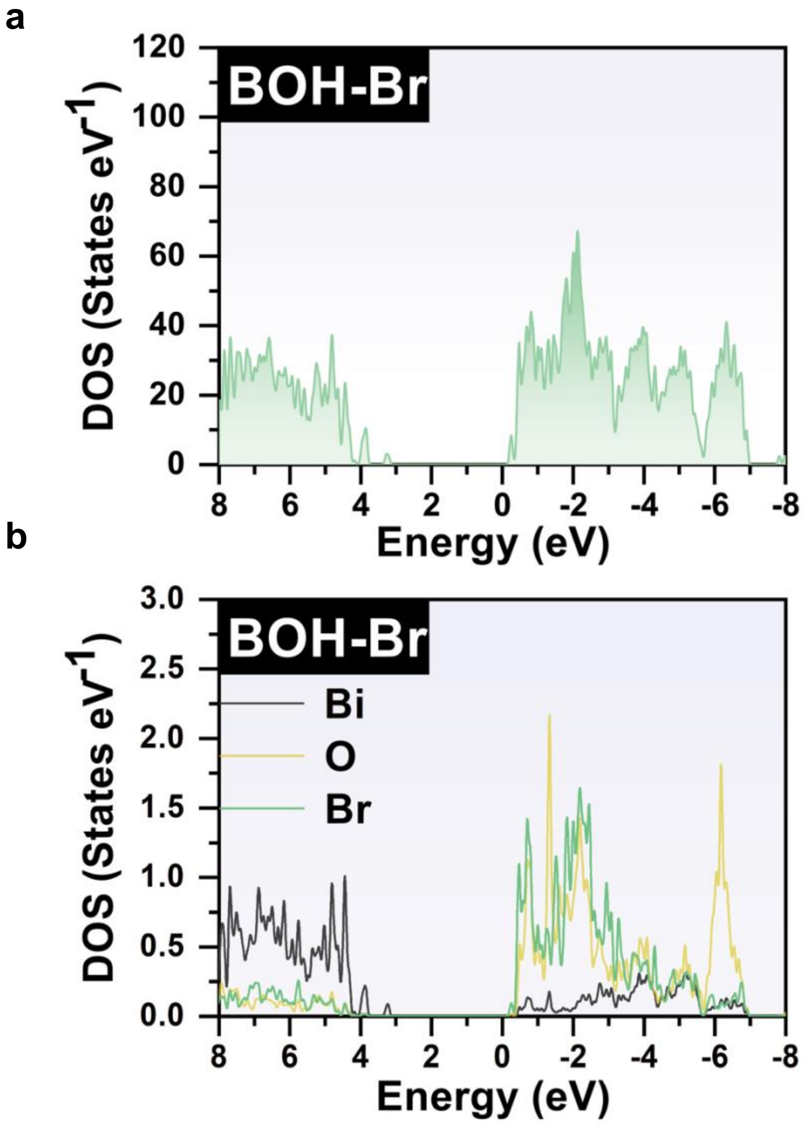

Supplementary Figure 39. The DOS calculation for BOH-Br. a The calculated density of state of BOH-Br. **b** The calculated partial density of state of bismuth, oxygen and bromine element for BOH-Br, respectively.

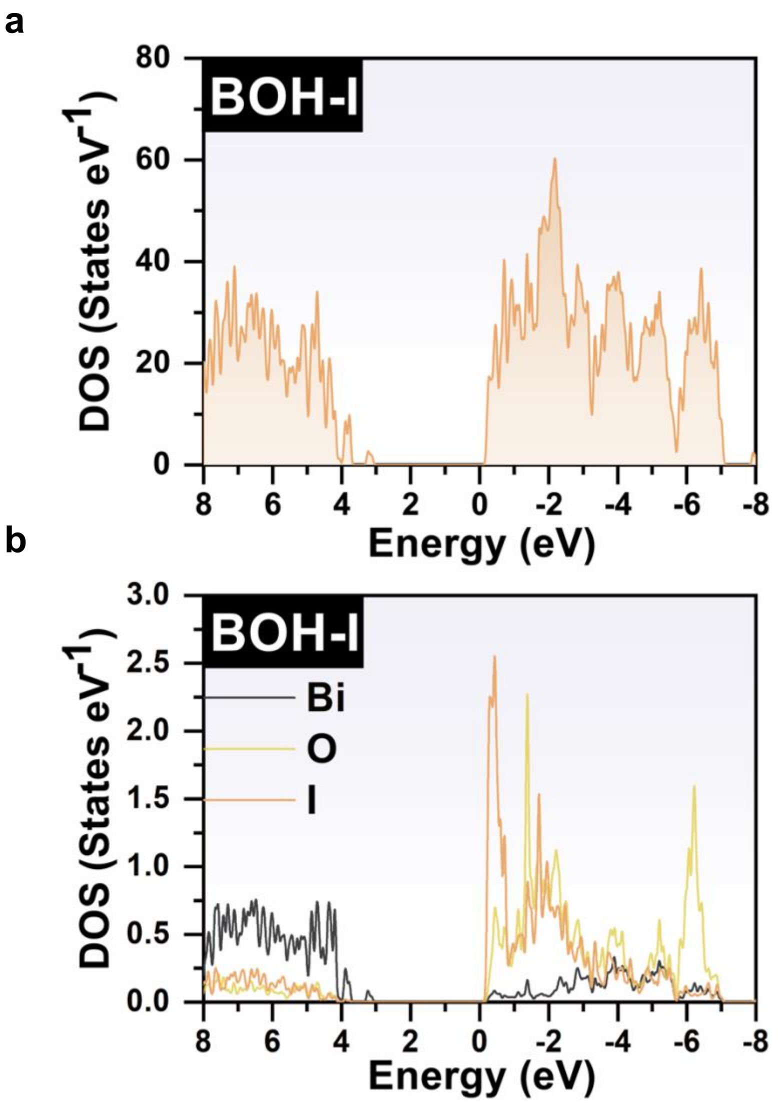

Supplementary Figure 40. The DOS calculation for BOH-I. a The calculated density of state of BOH-I. **b** The calculated partial density of state of bismuth, oxygen and iodine element for BOH-I, respectively.
